# Identification of Novel Regulators of Leaf Senescence Using a Deep Learning Model

**DOI:** 10.3390/plants13091276

**Published:** 2024-05-05

**Authors:** Chaocheng Guo, Zhuoran Huang, Jiahao Chen, Guolong Yu, Yudong Wang, Xu Wang

**Affiliations:** Shanghai Collaborative Innovation Center of Agri-Seeds, Joint Center for Single Cell Biology, School of Agriculture and Biology, Shanghai Jiao Tong University, Shanghai 200240, China; guocc212@sjtu.edu.cn (C.G.); 342937279@sjtu.edu.cn (Z.H.); 435224397@sjtu.edu.cn (J.C.); yuguolong@sjtu.edu.cn (G.Y.); yudongwang@sjtu.edu.cn (Y.W.)

**Keywords:** gene regulation network, single-cell transcriptome analysis, deep learning, transcription factors, leaf senescence

## Abstract

Deep learning has emerged as a powerful tool for investigating intricate biological processes in plants by harnessing the potential of large-scale data. Gene regulation is a complex process that transcription factors (TFs), cooperating with their target genes, participate in through various aspects of biological processes. Despite its significance, the study of gene regulation has primarily focused on a limited number of notable instances, leaving numerous aspects and interactions yet to be explored comprehensively. Here, we developed DEGRN (Deep learning on Expression for Gene Regulatory Network), an innovative deep learning model designed to decipher gene interactions by leveraging high-dimensional expression data obtained from bulk RNA-Seq and scRNA-Seq data in the model plant Arabidopsis. DEGRN exhibited a compared level of predictive power when applied to various datasets. Through the utilization of DEGRN, we successfully identified an extensive set of 3,053,363 high-quality interactions, encompassing 1430 TFs and 13,739 non-TF genes. Notably, DEGRN’s predictive capabilities allowed us to uncover novel regulators involved in a range of complex biological processes, including development, metabolism, and stress responses. Using leaf senescence as an example, we revealed a complex network underpinning this process composed of diverse TF families, including *bHLH*, *ERF*, and *MYB*. We also identified a novel TF, named MAF5, whose expression showed a strong linear regression relation during the progression of senescence. The mutant *maf5* showed early leaf decay compared to the wild type, indicating a potential role in the regulation of leaf senescence. This hypothesis was further supported by the expression patterns observed across four stages of leaf development, as well as transcriptomics analysis. Overall, the comprehensive coverage provided by DEGRN expands our understanding of gene regulatory networks and paves the way for further investigations into their functional implications.

## 1. Introduction

Complex traits are coordinated across diverse cell types and tissues by hormones, metabolites, and mechanical forces, aiming to generate a coherent plant response to the environment [1]. The key to unpicking what underpins these traits is to determine the gene expression that occurs, which is principally independent in each plant cell [2]. Gene expression is regulated by numerous factors, including transcription factors (TFs), epigenetic factors, and miRNAs [3]. Notably, the primary regulators of gene expression are transcription factors, which can bind specific DNA sequences and regulate the expression of downstream genes. Hence, gene regulatory networks (GRNs), which consist of nodes (TFs and their targets) and edges (the relationships of TFs and targets), link TF genes to target genes and are thus used to represent condition-specific interactions of genes and their regulators [4]. GRNs are a powerful tool for identifying the potential regulators and regulatory relationships of the development process and stress response [4,5,6]. An example GRN is a salt-responsive GRN built by the temporal transcriptional patterns of *Marchantia polymorpha* and *Arabidopsis thaliana*, contributing to the evolutionary divergence of land plants at a network scale, a process that could not be unpicked using the previous methods [7]. As this exemplifies, constructing an accurate GRN with a large number of nodes and high quality is one of the key tasks in systems biology [8].

There have been numerous methods for constructing GRNs with different biological components. Generally, TFs can recognize the specific binding sequences on the promoters of target genes, enabling them to represent protein–DNA interactions. For example, the AtRegNet database, which was updated in 2019, collects 1,638,778 direct physical interactions between TFs and the promoters of their target genes [9]. Based on TF binding motifs, the TF2Network can identify potential regulators or functionally related genes [10]. In addition, ConnecTF integrates different datasets, including TF–target binding and TF–target regulation, through a website on which users can obtain TF–target information [11]. Meanwhile, an integrative GRN (iGRN) uses seven input networks, including TF ChIP data, position weight matrices (PWMs), and co-expression (CoE) data, to build an integrative inference of a transcriptional network with 1,709,871 interactions via a supervised learning approach. However, it is limited to the evidence from public ChIP-Seq or DAP-Seq data, which cover a limited number of TFs existing in plants. Recently, the accurate construction of GRNs has become feasible due to the accumulation of abundant transcriptomic data in plants. Representative methods include GENIE3 [12], EXPLICIT [3], and WGCNA [13], which use gene expression data as inputs. For instance, EXPLICIT collects 24,545 RNA-Seq datasets within a model based on regression. It can infer transcriptional regulators for Arabidopsis genes and has high power in TF module detection [3]. However, the transcriptomic data that it utilizes are at the tissue level, which hinders the discovery of subtle but critical differences among cells. Recent technologies, such as single-cell RNA-Seq (scRNA-Seq), facilitate our understanding of the heterogeneity of different types of cells at a transcript scale, providing a higher resolution of cellular differences than bulk RNA-Seq [14,15,16]. While scRNA-Seq has only been performed in a few plant species, such as Arabidopsis [17,18], tomato [19], maize [20], and rice [21], it has been shown that scRNA-Seq data are powerful when utilized in GRN reconstruction in humans and mice [22,23,24]. Therefore, constructing an accurate GRN with scRNA-Seq data in plants seems a promising research pathway.

Compared to classical statistical tools, deep learning has recently exhibited an improved power to unpick complex biological processes in plants [25,26]. For example, PlantDeepSEA uses the results of ATAC-seq to build a model that predicts the open chromatin region (OCR), which can be further used to identify the *cis*-elements [25]. The neural network DENA, which was trained on direct RNA-Seq data of in vivo-transcribed mRNAs from wild-type and m^6^A-deficient *Arabidopsis thaliana*, can identify 90% of miCLIP-detected m6A sites in Arabidopsis [26]. However, the application of deep learning to multiple dimensions of expression data remains limited. Here, we designed a neural network called DEGRN (Deep learning on Expression for Gene Regulatory Network), which was trained on both bulk RNA-Seq and scRNA-Seq expression data. DEGRN obtained 3,053,363 high-quality interactions with a score > 0.5, containing 1430 TF genes and 13,739 non-TF genes. The neural network also showed greater power to recover TF regulators when compared with existing tools applied in plants. DEGRN provides a valuable resource for the inference of novel gene functions associated with each TF gene, encompassing a range of biological processes such as development, metabolism, and stress responses. The application of DEGRN has led to the discovery of previously unknown TFs that play a crucial role in the regulation of leaf senescence. For instance, we identified a transcription factor named MAF5, whose mutant exhibited premature leaf senescence, highlighting the crucial role of MAF5 in the process of leaf senescence. Our study expands our understanding of gene regulatory networks and paves the way for further investigations into their functional implications.

## 2. Results

### 2.1. A Deep Learning Model for Identifying TF and Target Interactions 

**Figure 1:**
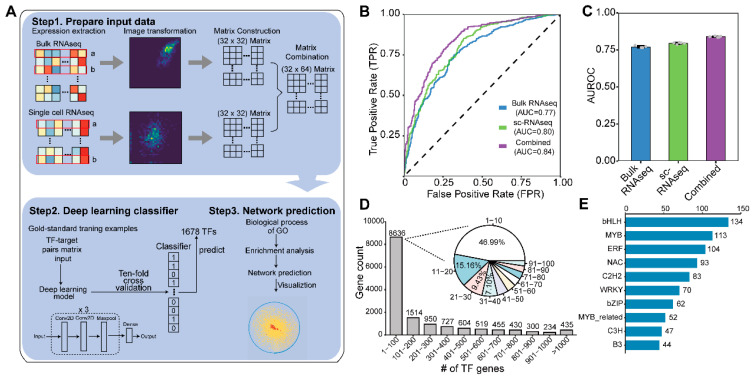
Overview of the establishment of DEGRN. (**A**) Process of building DEGRN. Firstly, gene *a* and gene *b* were obtained from single-cell RNA sequencing (scRNA-Seq) to be visualized into two dimensions of the matrix. When combining the two datasets, the newly produced matrix was used as input for DEGRN via a convolutional neural network (CNN). Thus, using a gold standard of TF–target interactions, we predicted 1678 existing transcription factors (TFs) via DEGRN, thus inferring the novel gene function of each TF and constructing the novel network with various TFs, targets, and functions. (**B**) Receiver operating characteristic (ROC) plot of three models based on bulk RNA-Seq, scRNA-Seq, and the combined data. (**C**) Average value of the ROC (AUROC) with five repeats for the above three models. (**D**) Distribution of a number of predicted TFs per target gene. (**E**) Number of TF families predicted by DEGRN.

When seeking to build a robust model for inferring the relationship between transcription factors (TFs) and their potential targets, the core of this process was to recover the diverse expression of TFs and their targets. To do so, we collected a bulk RNA-Seq expression dataset with 728 cultivars from 1001 Arabidopsis projects, which represents the expression diversity of most cultivars of Arabidopsis with diverse genotypes. Simultaneously, we collected a single-cell RNA-Seq (scRNA-Seq) dataset of root tissue in Arabidopsis, which can be characterized by the expression diversity of different tissues (cells) within an individual. The two datasets enabled us to explore the potential transcriptional regulation at the genome-wide level in Arabidopsis. Using these datasets, we built a convolutional neural network (CNN) model, named DEGRN (Deep learning on Expression for Gene Regulatory Network), containing six convolutional layers, one flattened layer, two dense layers, and a final “sigmoid” layer. For the inputs of DEGRN, the expression levels of gene *a* and gene *b* were separately extracted and transformed as a 2D histogram image (Figure 1A). The 2D image was used for a higher-dimensional matrix (such as 32 × 32) to fit the input of the CNN model. This method can improve the utilization of the expression data. When two separate datasets are generated, the two can also be jointly combined to form a larger matrix (example as above, 64 × 32) as the input of DEGRN (see Methods). The interaction of gene *a* and gene *b* was labeled as “1”, indicating that gene *a* regulated gene *b*, while “0” meant that gene *a* and gene *b* were not interacting. The output of the model was normalized from 0 to 1, where the closer the output was to 1, the more likely it was that interactions were present. With DEGRN, we trained a series of gene pairs from a gold-standard dataset previously built by iGRN, which contained 47 TF families and 11,460 interactions with an average of 11 interactions per TF [27]. To measure the performance of DEGRN, we used the area under the curve receiver operating characteristic (AUROC) as the metric on a scale of 0 to 1, with 1 being the most accurate.

We then used 70% of the gene pairs of the gold standard, which were randomly selected and used as a training dataset for the model’s training via deep learning, while the remaining 30% were used as an evaluation set. To measure the impact of expression datasets produced using different techniques, three different inputs were used to train our models. Firstly, the bulk RNA-Seq dataset was independently used to train the model, and the average value of AUROC was 0.770 (±0.0065). Secondly, the model trained with the scRNA-Seq dataset achieved a more efficient and higher AUROC value (0.795 ± 0.0050). Interestingly, the AUROC value of the model trained on either dataset alone was significantly lower than that of the model trained by combined expression (average AUROC_combine_ = 0.839 ± 0.0036), suggesting that the combination of bulk RNA-Seq and scRNA-Seq can better reflect transcriptional regulatory relationships (Figure 1B,C). Furthermore, ten-fold cross-validation (CV) with 20 repeats was used to evaluate the performance and stability of our model, in which the model was trained on 90% of the gene pairs and tested on the remaining 10%. Among those repeats, the average AUROC value was 0.85, indicating that the model performed well among those datasets (Table S1). In consideration of the fact that the dimensions may have impacted the accuracy, we assessed the effect of changing the dimensions of the expression matrix (i.e., 16 × 16, 32 × 32, and 64 × 64) on the AUROC of our model for the classification of interaction (Figure S1). The result suggested that 32 × 32 was the optimal size for the model. Thus, using both bulk RNA-Seq and scRNA-Seq data, and with a 32 × 32 expression matrix, DEGRN was built for further analysis. 

By applying DEGRN to predict the interactions of 1678 TFs and 29,182 non-TF genes, we obtained a comprehensive landscape of transcriptional regulation with 3,053,363 interactions, containing 1430 TF genes and 13,739 non-TF genes. Among those interactions investigated, most target genes (62.86%) were targeted by fewer than 100 TF genes, while 435 (3.17%) genes were potentially regulated by more than 1000 TFs, suggesting the versatility of these genes. Of note, 46.99% of the genes in the “1–100” group were targeted by fewer than 10 TFs (Figure 1D). Furthermore, our study encompassed a wide array of TF families, including the bHLH family, MYB family, ERF family, and NAC family (Figure 1E). By investigating members from these diverse TF families, we obtained a comprehensive understanding of the regulatory landscape underlying complex biological processes. 

### 2.2. Performance across Diverse Datasets 

To test whether DEGRN had significant predictive power in other datasets, we selected four public transcriptome datasets of scRNA-Seq for different tissues and one transcriptome dataset of 1001 Arabidopsis, to generate eight test datasets for further evaluation. To better assay the power of DEGRN, two strategies were used. Firstly, we used the above eight datasets as the input data of DEGRN for model training and thereby obtained eight models for these eight datasets. We found that most models (all except for Dataset 8) had an AUROC value of more than 0.8, which was poor (Figure S2A,B). We reasoned that this result was caused by the limited number of samples in the expression data. The comparable performance of the above eight models with DEGRN implied the power of the neural network when dealing with diverse datasets. Secondly, we used the above eight datasets as the evaluation data of DEGRN, to generate predictive data, the AUROC, and the F1 score between the observed data and predictive data. Despite the low AUROC values and F1 scores in the above datasets, DEGRN still had modest power (AUROC > 0.6 and F1 score > 0.5) to predict the interactions among these diverse datasets (Figure S2C,D). 

### 2.3. Validation of TF–Target Interactions according to the Experimental Evidence

**Figure 2:**
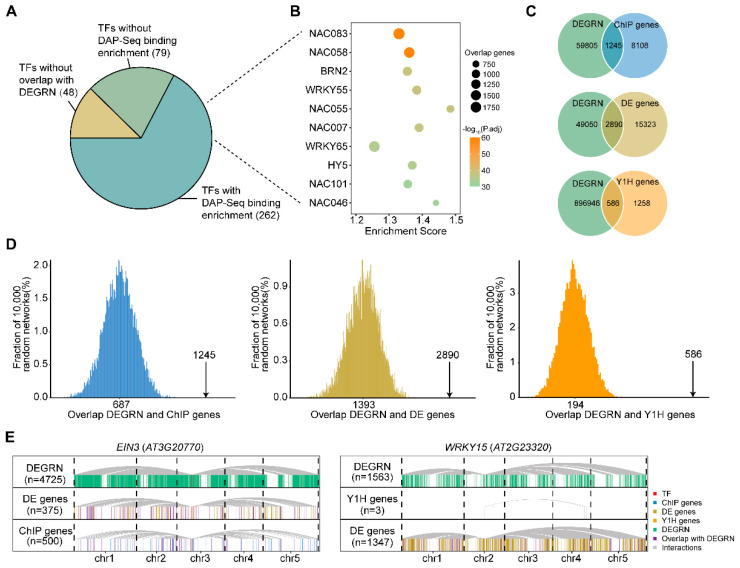
DEGRN was supported by experimental evidence. (**A**) Results of our comparison and enrichment analysis between TF–target interactions from DAP-Seq and DEGRN. (**B**) Top 10 TFs enriched with DAP-Seq. (**C**) Overlap of TF–target interactions between DEGRN, ChIP genes, DE genes, and Y1H genes. (**D**) Histogram plots of the expected overlap between DEGRN and the three sets of experimental evidence based on 10,000 randomized networks. The black arrows represent the observed overlap, and the number represents the expected overlap of 10,000 randomized networks by chance. The *p*-value was defined as the number of expected overlaps by chance exceeding the observed overlap divided by 10,000. (**E**) Examples of overlaps of *EIN3* (*AT3G20770*) and *WRKY15* (*AT2G23320*) are shared by DEGRN and sets of experimental evidence. The red bars represent the selected TFs. The blue, brown, yellow, and green bars represent the targets obtained by ChIP data, DE data, Y1H data, and DEGRN, respectively. The purple bars represent the overlap targets, which were covered by DEGRN and experimental evidence. The gray lines represent the interactions between TFs and their targets.

To evaluate the accuracy of the interactions predicted by DEGRN, we utilized an independent DAP-seq dataset containing 387 TFs with in vitro physical binding to the promoters of their target genes. The interactions of our model were compared with the DAP-seq binding target gene list, thus evaluating if there was significant overlap between the two. Among those 387 TF genes, 88.11% (341) were found to overlap between the dataset of DAP-Seq and DEGRN, while 46 TF genes did not overlap. Interestingly, 262 (or 67.70% of the) TF genes with target genes in DEGRN showed significant enrichment for DAP-Seq binding among their target genes (*p* < 0.05 after Benjamini–Hochberg (BH) adjustment) (Figure 2A). For example, the top 10 most enriched TFs comprised *NAC083*, *NAC058*, *BRN2*, *WRKY55*, *NAC055*, *NAC007*, *WRKY65*, *HY5*, *NAC101*, and *NAC046*, in which *NAC083* shared an overlap of 1991 targets with the DAP-Seq data (Figure 2B; Table S2). Those results suggested that DEGRN has good potential in predicting the interactions of TF genes and their target genes. 

Next, we used three external datasets to evaluate the performance of DEGRN. The first dataset comprised TF ChIP-bound genes (called “ChIP genes”) for 24 different TFs, reflecting primarily direct binding in vitro (9353 interactions selected for the top 500 per TF), while the second dataset contained a set of 23 differentially expressed genes (called “DE genes”) after TF perturbation (18,213 interactions). The third dataset covered 1115 Y1H interactions directly reflecting interactions in vitro (called “Y1H genes”). The performance of DEGRN was measured by examining the overlaps, to determine if those were more significant than the expected overlaps generated randomly by DEGRN. In this way, an overlap of 1245 interactions (13.31%) was detected between DEGRN and ChIP genes, which was a 1.81 higher overlap than that expected by chance (687) (Figure 2C,D; Table 1). The DE genes displayed an overlap of 2890 edges (15.87%), with an enrichment fold of 2.07 (2890/1393). The third dataset, of Y1H genes, contained the minimum number of interactions, with us only detecting an overlap of 586 interactions (31.78%), which showed a higher enrichment (3.02-fold enrichment) than those for the other two datasets (Figure 2C,D). For example, the TF gene *EIN3*, which is involved in ethylene responses, was predicted with 4725 targets by DEGRN. Among those targets, we found that 43.47% (163) and 21.80% (109) of DE genes and ChIP genes overlapped with DEGRN, respectively, implying a high similarity between these two datasets. Similarly, the TF gene *WRKY15*, with 1563 targets by DEGRN, was found to overlap in 100% (3) and 12.47% (168) of targets with DEGRN, respectively (Figure 2E). Overall, these results offered support for the interactions of DEGRN in the form of experimental evidence. 

### 2.4. Inference of Potential Gene Functions for TF Genes

**Figure 3:**
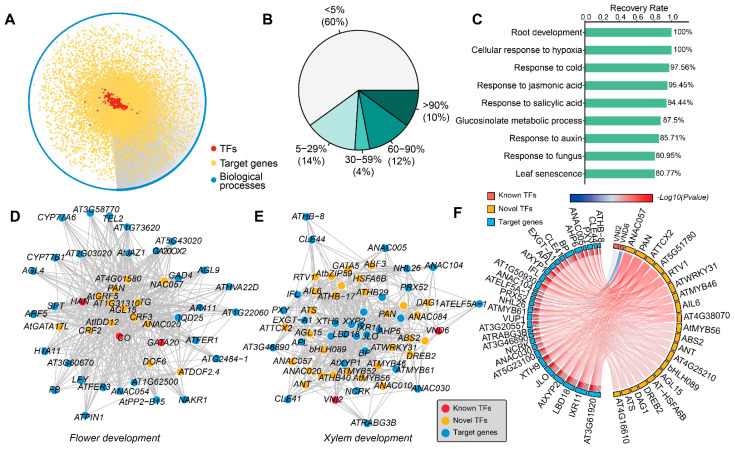
The inference of novel gene functions by DEGRN. (**A**) Overview of our inference of novel gene functions with DEGRN. The red dots in the center represent the TFs; the yellow dots represent the targets; the blue dots represent the GO terms of biological processes. (**B**) Recovery rate of known TFs per biological process (BP) term in Gene Ontology (GO). (**C**) Examples of BPs with high recovery rates. (**D**,**E**) show examples of networks of flower development and xylem development, respectively. (**F**) Examples of different TFs interacting with the common target genes within the same BP. The red bars represent the known TFs, while the yellow bars are the novel TFs obtained by DEGRN. The blue bars represent the common target genes. The colors of the line represent the −log_10_ value (*p*-value).

Using the above interactions, which were supported by functional research, the gene function of each TF was inferred via Gene Ontology (GO) enrichment analysis of its corresponding targets (Figure 3A; see Section 4.4). The GO terms with a general process were filtered out, while experimental biological process (BP) annotations were kept. If a term of a BP was enriched among the target genes, the corresponding TF gene was thought to be involved in this BP. The enriched terms of unannotated TFs were thought to represent a novel functionality. Among the 1424 TF genes predicted in DEGRN, 80.90% (1152) of TFs with known experimental BP annotations could be used to evaluate the accuracy of the validation of known functions, while the remaining TFs without any experimental BP annotations could be inferred as representing novel functions. As a result, 40% of known BPs were recovered with a rate > 5%, with an average of 23.51% of known regulators recruited (Figure 3B). Remarkably, there were several processes with a recovery rate higher than 80%, including root development (100%), response to hypoxia (100%), response to cold (97.56%), response to jasmonic acid (95.45%), response to salicylic acid (94.44%), glucosinolate metabolic process (87.50%), and respond to auxin (85.71%) (Figure 3C). Unfortunately, the terms related to seed development were not recovered, such as seed development (GO:0048316), seed coat development (GO:0010214), and seed germination (GO:0009845), which may have been due to the lack of seed tissues in our datasets. 

Based on the rule of guilt by association, the enrichment of target genes of TFs can be inferred to represent novel gene functions. Considering this, we found that there were abundant novel TFs predicted to be involved in the development of the plant, which had not been reported previously. For example, the most enriched term related to development was root development, which contained 1247 TFs, and 38 TFs were known regulators of root development (Figure S3A). Examples of these TFs were *AIL6* [28], *LRP1* [29], *ATMYB61* [30], *AT5G42700* [31], *ATWKRY9* [31], and *MGP* [32]. Likewise, seventeen TFs were responsible for flower development, of which three TFs (*CO*, *HAN*, and *GATA20*) had been reported previously [33,34] (Figure 3D). Meanwhile, 18 TFs were identified for leaf development, such as *bZIP59*, *MYB83*, *ANAC081*, and *DOF6* (Figure S3B). Furthermore, there were three TFs involved in anther development: *ARF5*, *DOF3.4*, and *AT4G29000* (Figure S3C). Meanwhile, four hundred and fifty-six TFs were related to pollen development, of which eight TFs were known regulators: *REM35*, *bHLH010*, *LRP1*, *AT3G10470*, *AMS*, *DREB2*, *ABS7*, and *ATWRKY43* [35,36,37] (Figure S3D). Otherwise, the TFs predicted to be involved in different aspects of development were explained by the significantly enriched target genes of the same BP. For example, one hundred and nine TFs were associated with xylem development, of which two (*VNI2* and *VND6*) are known regulators of xylem development [38,39] (Figure 3E). All of these TFs contained the common target genes involved in xylem or vascular development, including *IRX11*, *AtXYP2*, *JLO/LBD30*, *LBD18*, *BP*, *AHP6*, *VUP1*, *XTH9*, and *EXGT-A1/XTH4* [40,41,42,43,44,45] (Figure 3F). 

### 2.5. Prediction of Biological Processes Related to Metabolism and Stress Responses 

**Figure 4:**
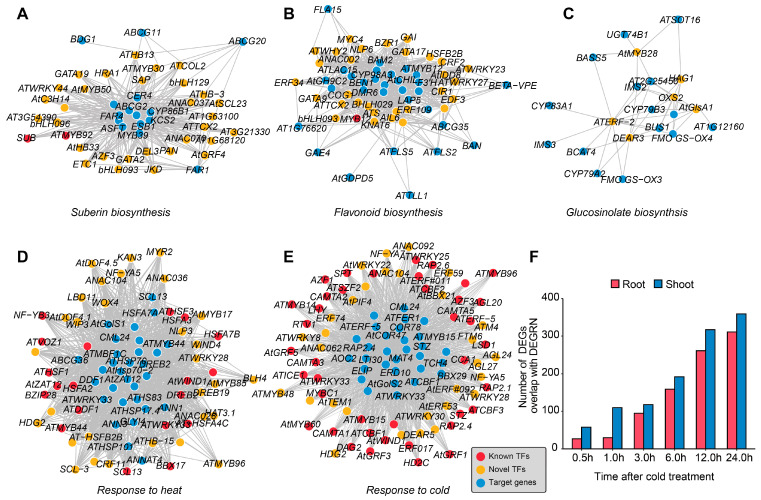
Prediction of the pathways related to metabolism and stress responses. (**A**–**C**) show three networks associated with metabolism, as predicted by DEGRN: suberin biosynthesis, flavonoid biosynthesis, and glucosinolate biosynthesis. (**D**,**E**) show examples of novel networks associated with abiotic stresses: heat stress and cold stress. The red dots represent the known TFs that were previously reported; the yellow dots represent the novel TFs predicted by DEGRN; the blue dots represent the target genes obtained by DEGRN; and the grey lines represent the correspondence of a TF and its target genes, which were enriched in the BPs. (**F**) Overlap genes between differentially expressed genes (DEGs) and novel TFs predicted by DEGRN. The red bars represent root tissues, while the blue bars represent shoot tissues.

In addition to the developmental processes, the novel TFs related to metabolism were also investigated. As a result, three hundred and eighty TFs were uncovered for suberin biosynthesis, of which two TFs (*SUB* and *MYB92*) had been reported for suberin biosynthesis [46,47] (Figure 4A). As for flavonoid biosynthesis, 882 TFs were able to regulate this process, though only *ATMYB12* had been shown to participate in this process [48], suggesting that DEGRN may perform well at inferring novel gene functions related to basic metabolism (Figure 4B). Additionally, six novel TFs were identified for glucosinolate biosynthesis: *ATERF-2*, *DEAR3*, *AtGlsA1*, *AT3G06410*, *OXS2*, and *HAG1* (Figure 4C). Meanwhile, there were four novel TFs, *GNC*, *ESE3*, *CGA1*, and *AT1G12890*, responsible for fatty acid biosynthesis, which had been not reported previously (Figure S4A). 

At the same time, we identified novel TFs for processes related to stress responses. Of note, for the terms related to cold stress and heat stress, we obtained a large number of TFs, including 1258 TFs for heat and 1375 TFs for cold, which had the potential to regulate these processes (Figure 4D,E). Among these TFs, 40 had been reported to respond to cold, including the key TF genes *CBF1*, *CBF2*, and *CBF3* [49,50]. Similarly, nineteen TFs were known for the heat response, of which six belong to the HSF family, namely, *HSFA2*, *HSFA7B*, *HSFA3*, *HSFA4C*, *HSF1*, and *HSF3* [51,52,53]. To validate the accuracy of these, we compared the network of cold responses with public time-series transcriptome data after cold treatment. We found that the overlap between differentially expressed genes (DEGs) and DEGRN increased gradually with time after cold treatment, no matter whether in root or shoot (Figure 4F). When combining these times, we identified 736 TFs, consisting of 53.53% of the genes identified by DEGRN, which were differentially expressed at a time point, suggesting a large overlap between DEGRN and the previous study that produced these data. Moreover, there were 38 TFs associated with response to UV, 102 TFs related to salt stress, and 15 TFs involved in metal ions (Figure S4B–D). 

### 2.6. DEGRN Accelerates the Investigation of Leaf Senescence 

**Figure 5:**
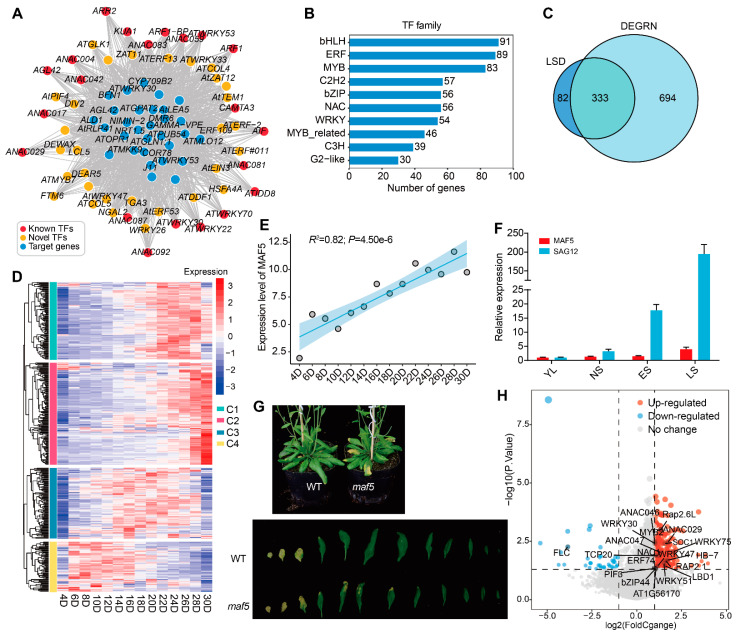
The application of DEGRN in leaf senescence. (**A**) Novel network of leaf senescence predicted by DEGRN. The red dots represent the known TFs; the yellow dots represent the novel TFs obtained by DEGRN; the blue dots represent the target genes; and the grey lines represent the correspondence of a TF and its target genes. (**B**) Distribution of the TF family involved in leaf senescence. (**C**) Overlap between TFs identified by DEGRN and senescence-associated genes (SAGs) obtained from Leaf Senescence Database (LSD) 3.0. (**D**) Expression of novel TFs in the lifespan of Arabidopsis from public transcriptome data. (**E**) Linear regression of MAF5 during the process of leaf senescence. (**F**) Relative expression of *MAF5* at four stages of leaf development. YLs, young leaves; NS, fully expanded mature leaves without senescence symptoms; ES, early senescent leaves with <25% leaf area yellowing; LS, late senescent leaves with >60% leaf area yellowing. (**G**) Phenotype within wild-type (WT) and mutant *maf5*. (**H**) Fold change of the known TFs identified by public population-scale transcriptome data for 1001 Arabidopsis. The differentially expressed genes (DEGs) were identified between the accessions with the lowest and highest expression levels of *MAF5*.

Leaf senescence is a complex biological process, which can be regulated by various factors, such as hormones, circadian rhythm, and stress [54,55]. Our network has the potential to identify novel TFs involved in leaf senescence, and we tested this potential out. As a test case, a large number of TFs with predicted leaf senescence (1027) were extracted, of which 21 TFs were known to respond to leaf senescence (Table S3; Figure 5A). Among those known TFs, ten TF genes belong to NAC transcript factors, such as *ANAC087*, *NAC059*, *NAC081*, *NAC017*, *NAC042*, *NAC029*, *NAC092*, and *NAC053* [56,57,58,59], and there were four WRKY genes known for leaf senescence: *WRKY22*, *WRKY30*, *WRKY53*, and *WRKY70* [60,61]. WRKY70, a negative senescence regulator, can cooperate with WRKY54 to regulate leaf senescence via a salicylic-acid-dependent pathway [61]. In contrast, WRKY53, which is induced at an early stage of leaf senescence, acts as a positive regulator of senescence [62]. It was reported that *NAC017* negatively regulated leaf senescence together with *ANAC090* by suppressing the reactive oxygen species (ROS) response, which was referred to as a “NAC troika” [58]. Among the remaining 1026 TF genes, the bHLH TFs were the most abundant family, with 91 genes predicted to be responsible for leaf senescence, followed by the ERF (89 genes) and MYB (83 genes) families (Figure 5B). We then compared the novel TFs identified by DEGRN with the potential leaf-senescence-related genes (SAGs) collected from the Leaf Senescence Database (LSD), which compiles 3852 genes supported by genetic or genomic evidence or microarray data [63]. After filtering out the non-TF genes from the LSD, the remaining 415 TFs displayed an overlap of 333 genes (80.24%), with a fold enrichment of 2.14, indicating high performance in the network of leaf senescence (Figure 5C).

To evaluate the potential role of novel TFs in leaf senescence, we investigated the expression pattern of these novel TFs through the lifespan of Arabidopsis leaves. To do so, with public transcriptome data (GSE43616) from the GEO database, we were able to investigate whether these novel TFs were involved in or regulated during the senescence stage. As shown in Figure 5D, we found that these TFs could be classified into four groups, named C1–C4. The groups C1 and C2 were highly expressed in the late developmental stage from 20D to 30D, suggesting a potential role of these TFs in the late stage. Previous studies showed that many genes involved in auxin biosynthesis were up-regulated during age-dependent senescence [64,65]. We identified serval ARF genes in groups C1 and C2, such as *ARF4*, *ARF7*, *ARF9*, *ARF16*, and *ARF17*, suggesting that auxin may function in the process of leaf senescence [65]. Group C4 showed high expression in early development from 4D to 14D, while group C3 increased among the mid-stage from 14D to 22D. 

We also found a TF named *MAF5*, belonging to C1, which showed strong linear regression with a significant *p*-value (*R*^2^ = 0.82, *p*-value = 4.50 × 10^−6^), suggesting its potential role in leaf senescence (Figure 5E). To validate this function of MAF5, we assayed its expression in the four stages of leaf development, including young leaves (YLs) and fully mature leaves without senescence symptoms (NS), in early senescence (ES), and in late senescence (LS) (Figure 5F). The results indicated that the relative expression of *MAF5* gradually increased with the progression of senescence, which showed a similar trend to the leaf senescence marker gene *SAG12* (Figure 5F). Similarly, we observed little leaf decay in the mutant *maf5* compared to the wild-type *col-0* (Figure 5G). To investigate the mechanism of *MAF5* in leaf senescence, we evaluated transcriptional changes through the public transcriptome of 1001 Arabidopsis. We defined the samples with high expression of *MAF5* as *MAF5^+^*, while the samples with low or no expression were defined as *MAF5*^−^. With the criteria of “|LogFC| > 1 and *p* value < 0.05”, we identified that 20 known genes collected from LSD were differentially expressed between *MAF5*^−^ and *MAF5^+^*, including *ANAC029*, *ANAC046*, *bZIP44*, *WRKY75*, and *LBD1* (Figure 5H). This result suggested that the reduction in *MAF5* caused alterations in a variety of leaf senescence genes. Finally, we investigated the functions of these DEGs via GO and KEGG enrichment analyses. The results showed that most DEGs may be involved in these terms related to “response to salicylic acid”, “response to oxidative stress”, “response to hypoxia”, and “plant hormone signal transduction” (Figure S5A,B). Thus, we speculated that phytohormones may play a crucial role in the pathway of MAF5 with leaf senescence. However, the mechanism needs to be validated through further experiments. 

### 2.7. The Performance of DEGRN Compared with EXPLICIT, iGRN, and AtRegNet

To evaluate the robustness of DEGRN, three methods previously reported, EXPLICIT [3], iGRN [27], and AtRegNet [9], were compared with DEGRN. Firstly, we compared the completeness of the whole TFs across different methods. For the number of TFs analyzed, DEGRN obtained 3,053,363 interactions covering 1430 TFs, which was higher than AtRegNet (585) and comparable with iGRN (1491) and EXPLICIT (1679). However, the average number of interactions per TF in our model (2135) was greater than those in iGRN (1146) and EXPLICIT (584). DEGRN also showed the best performance in the median number of interactions per TF (1876), implying the continuity of DEGRN (Table S4). The details per TF family were as follows: the bHLH family was predicted by iGRN with the highest members (142) and an average of 1345 interactions for each member, followed by EXPLICIT (140 members and 588 interactions per member), DEGRN (126 members and 2160 interactions per member), and AtRegNet (32 members and 861 interactions per member). Although the number of members predicted in DEGRN was a little lower, the average interactions in our model were 1.61 and 3.67 times greater than in iGRN and EXPLICIT (Table S5). Furthermore, DEGRN outperformed on several more TFs than the other three methods. For example, the C3H family, containing 13 members, was predicted to have the greatest average number (2036) of interactions, which was five and two times more than iGRN (431) and EXPLICIT (977), respectively. AtRegNet did not cover one member of the C3H family. Next, for each TF gene in the C3H family, we compared the overlapped target genes of three of the methods: iGRN, EXPLICIT, and DEGRN. We found that 61.53% of the 13 TFs showed a greater overlap in genes between DEGRN and iGRN (or EXPLICIT) than between iGRN and EXPLICIT. These results demonstrated that our model was able to obtain better results than the other three methods on TF target pairs.

Secondly, the gene functions were compared of TF genes consisting of target genes with the same GO BP annotations. According to the TF pairs from the other three methods, the predicted BP was calculated for each TF. To measure the performance of different methods, the power was defined as the number of known regulators previously reported. We found that the BPs were associated with nine types of abiotic stresses: cold stress (GO:0009409), heat stress (GO:0009408), salt stress (GO:1902074), hypoxia stress (GO:0001666), osmotic stress (GO:0006970), oxidative stress (GO:0006979), ethylene stress (GO:0009723), jasmonic acid stress (GO:0009753), and salicylic acid (GO:0009751). DEGRN obtained 13.11 known regulators on average, which was slightly higher than iGRN (12.67), EXPLICIT (12.11), and AtRegNet (7.56) (Table S6). In particular, DEGRN significantly outperformed the other methods in several BPs, covering cold stress, salt stress, hypoxia stress, ethylene stress, and salicylic acid stress. For example, DEGRN obtained 12 known TFs that were involved in cold stress, exceeding the other methods. Examples of known TFs associated with cold stress covered *NLT6*, which is induced by cold and, in turn, induces a small group of cold-inducible PR (pathogenesis-related) genes to elicit pathogen resistance [66]. Overall, these results suggest that DEGRN outperforms the other methods when predicting the novel gene functions of TF genes. 

## 3. Discussion

Gene regulatory networks (GRNs) are powerful tools for understanding the complexity, functionality, and pathways of biological systems, including development, metabolism, and stress responses [7]. Since the advent of high-throughput technologies in biology in the late 1990s, reconstructing GRNs has stood as a central computational problem in systems biology [67]. Recently, increasing evidence has begun to suggest that deep learning is a useful method for addressing various biological problems [25,68,69]. However, there are few published works on deep learning used to study GRNs in plants. Hence, we used the expression data of bulk RNA-Seq and scRNA-Seq to develop a deep learning model (DEGRN) for inferring the interactions of TFs and their target genes. There are a set of popular newly developed methods in deep learning, such as iGRN [27] and TFBSnet [70]. However, most of these methods depend on the limited ChIP binding site data of specific TFs. By overcoming that limitation, and benefitting from the improved power of deep learning and large-scale data, we can screen almost all potential transcription factors for interactions with candidate target genes. Accordingly, the DEGRN used here is not limited to specific TF binding sites, for which data are not easily obtained, but instead utilizes transcriptome data available in public databases. However, due to the specificity of gene expression in different tissues, it was found that several TFs were not predicted for any interactions in our data because of their low or even absent expression. We propose that merging expression data on genes in more tissues may have a great effect on the universal construction of GRNs.

Compared with traditional bulk RNA-Seq data, which are mixed for different cell types, single-cell RNA-Seq (scRNA-Seq) is a powerful tool for identifying and quantifying transcriptional activity at a single-cell resolution. Previous studies showed that scRNA-Seq can perform well in plants, and it offers great advances in identifying transcriptional activity in individual cells, constructing development trajectories, and detecting novel cell identity markers [16,20,71]. In our study, we used expression data from scRNA-Seq (containing 17,290 cells in Arabidopsis roots) for GRN construction using deep learning. The model built with expression data from scRNA-Seq outperformed that built on expression data from bulk RNA-Seq, implying the potential of scRNA-Seq in gene regulation. Furthermore, all three models showed high AUROC values (greater than 0.75), suggesting that expression is an important feature when constructing models with which to investigate transcriptional regulation. We also hypothesized that scRNA-Seq would contain additional transcriptional information within a cell type or a tissue compared to the scant bulk RNA-Seq and thus would provide a higher-resolution GRN, thereby producing more robust results. Accordingly, our results suggested that using such large-scale expression data from scRNA-Seq could make it more feasible to explore the secrets of transcriptional regulation in plants. With technological development, more and more non-model species can be investigated for transcriptional regulation via scRNA-Seq, such as peanut [72] and crantz [73]. There is a possibility that DEGRN could be applied to these non-model species. However, it is important to note that further validation and testing are necessary to assess the feasibility and effectiveness of DEGRN in these species. 

Inferring novel gene functions or novel regulators of TF genes is the key role of a GRN. Through these high-quality interactions of DEGRN, several novel regulators were identified for abundant biological processes in Arabidopsis, including development, metabolism, and stress responses. We used 1152 TFs to evaluate the recovery of known BPs and infer novel functions, while 272 TFs were inferred to have novel functions without any experimental BP annotations. The highly accurate recovery of known regulators implies that DEGRN performs well in inferring TF gene functions. Notably, we focused on biological processes related to development, metabolism, and stress responses. Examples of development include root, flower, leaf, xylem, anther, and pollen development. Among these processes, we obtained many typical genes involved in development. These typical known regulators included *CO*, *REM35*, *bHLH010*, and *LRP1* [34,35,36]. For stress responses, we found that key genes from the CBF family and HSF family were responsible for cold and heat stress, respectively [52,74]. These results suggest that DEGRN is a powerful tool for inferring novel gene functions for TFs and thus constructing a more complete landscape of transcriptional regulations. 

A complicated biological process requires a more accurate network in order to explore its regulations. Here, we used the example of leaf senescence to display the performance of DEGRN in terms of its power to unpick complicated processes. Leaf senescence is known to be regulated by multiple factors, such as environmental factors (circadian rhythm, light) and genetic factors (epigenetic regulation, transcriptional regulation) [55]. We obtained 1027 TF genes related to leaf senescence, indicating that leaf senescence is highly complex and regulated by multiple TF genes. Among these TFs, 21 TFs were shown previously to regulate this process via various pathways, containing multiple members of the NAC family and WRKY family. For instance, NAC083 (VIN2) integrates ABA-mediated abiotic stress signals into leaf aging by regulating a subset of *COLD-REGULATED* (*COR*) and *RESPONSIVE TO DEHYDRATION* (*RD*) genes [75]. The MADS-box gene, namely FYF, acts as a repressor controlling floral organ senescence and abscission in Arabidopsis, while the transgenic plant 35S: FYF delays leaf senescence [76]. Apart from this, 415 senescence-associated TFs were obtained from the LSD 3.0 database, which collects SAGs (senescence-associated genes) from various species through multiple methods, and 80.24% (333) of TFs were uncovered using DEGRN, demonstrating the reliability of the predictions from DEGRN. Thus, transcriptome data for the lifespan of Arabidopsis leaves showed that novel TFs related to leaf senescence were dynamically changed during senescence. Our analysis of the random network indicated that these novel TFs were highly associated with leaf senescence. Overall, these findings suggest that DEGRN has potential to be used for exploring complex biological processes and that it may uncover interactions that are valuable for transcriptional regulation.

## 4. Materials and Methods

### 4.1. Gene Expression Data and Model Construction

To explore as many transcriptional regulators as possible, we selected bulk transcriptome data with 728 cultivars from 1001 Arabidopsis with the ID “GSE80744” in the Gene Expression Omnibus (GEO) database, which contains abundant transcriptional information at the population level. The scRNA-Seq data were obtained from the Single Cell Expression Atlas with ID E-GEOD-141730, representing 17,290 cells. For these datasets, the transcripts per million (TPM) expression value was calculated for each gene. 

The structure of DEGRN is composed of 6 convolutional layers and 3 max-pools. To build DEGRN, the gold standard of TF–target interactions was collected from a previous report [27]. For each gene pair, the gene expression data for the TF and its target were extracted from bulk RNA-Seq and scRNA-Seq, respectively. Next, due to the low expression and many zero values in scRNA-Seq, the expression data were normalized using log transformation. Then, we used the function “histogram2d” of Python to generate 2D histogram images for each gene pair with the parameter “bins = 32”, which produces a 32 × 32 matrix representing the relationship of the gene pair. To train the model, 70% of the whole data were selected as training data, while the rest were used as the testing data. For the models built solely from bulk RNA-Seq or scRNA-Seq, the input shape of the convolutional neural network (CNN) was set as 32 × 32. When combining the two datasets, the input shape of the CNN was set as 64 × 32. To evaluate the stability of DEGRN, 10-fold cross-validation (CV) was conducted with 20 repeats. The indicators related to the performance of the models were calculated as follows:

$$TPR=\frac{TP}{TP+FN}$$

$$FPR=\frac{FP}{FP+TN}$$

The true positive (TP) was defined as the interactions that exist in DEGRN and exist in the gold standard. The true negative (TN) was defined as the interactions that do not exist in DEGRN or the gold standard. The false positive (FP) was defined as the interactions that exist in DEGRN but not in the gold standard, while the false negative (FN) was defined as the interactions that do not exist in DEGRN but exist in the gold standard. To evaluate the performance of the models constructed by the different strategies, we selected the AUROC values as the criteria, which refer to the area under the receiver operating characteristic (ROC) curve drawn by the TPR and FPR. A set of 30,536,306 interactions were first predicted by DEGRN. We selected the top 10% of the whole interactions for further analysis with a probability of 0.72. With this threshold, we saw that the true positive rate (TPR) was 0.52, which was 4.3 times higher than the false positive rate (FPR). The code is available from https://github.com/guocc212/DEGRN (accessed on 1 January 2022).

### 4.2. Validation of DAP-seq and Experimental Results

The DAP-seq data (version 4) of TF–target gene pairs were downloaded from the Plant Cistrome Database, which contains 3,685,526 interactions [77]. For each TF, its target genes from DAP-seq were compared with the predicted target genes of DEGRN, using a hypergeometric distribution to determine if the two datasets significantly overlapped [3]. 

To validate the predicted network from DEGRN, three independent experimental datasets were used. The first dataset comprised the 9353 TF–target gene pairs of 24 different TF-ChIP data, which were collected from the literature (Table S7). To ensure credibility, only the top 500 target genes for each TF were considered. For TF perturbation, a set of DE genes, containing 18,213 interactions, was obtained from the literature on 23 TF genes [10]. Meanwhile, a set of 1844 Y1H interactions was collected from the literature (Table S7). Thus, the overlap of DEGRN and the above three datasets was defined as the number of interactions that were both present in DEGRN and experimental networks. The value of fold enrichment between two networks was defined as the number of interactions that were present in both networks divided by the number of interactions expected by chance. The interaction by chance was calculated as the average of the interactions from the permutation test with 10,000 repeats. The significance of the permutation test was defined as the number of times that the overlap of the random network was greater than the real overlap, divided by 10,000. 

### 4.3. Evaluation of DEGRN across Diverse Datasets

To assess the suitability of DEGRN, we selected five additional datasets for this study, including four single-cell transcriptome datasets of different tissues and one population-scale transcriptome dataset with 144 accessions of 1001 Arabidopsis [16,17,78,79]. The four single-cell transcriptome datasets were downloaded from the Single Cell Expression Atlas with IDs E-GEOD-161332, E-GEOD-121619, E-GEOD-123013, and E-MTAB-11006. The population-scale transcriptome data were downloaded from the GEO database with ID GSE43858. To better assay the effect of sample size and tissues, we constructed eight datasets for further analysis. Dataset 1 contained the scRNA-Seq data of E-GEOD-121619 and bulk RNA-Seq of GSE80744, while Dataset2 was mixed with scRNA-Seq data of E-GEOD-123013 and bulk RNA-Seq of GSE80744. Dataset 3 contained E-GEOD-161332 and GSE80744, while Dataset 4 contained E-MTAB-11006 and GSE807744. Datasets 5–8 were set up with the other bulk RNA-Seq (GSE43858) replacing GSE80744. The details of these databases are listed in Table S8. Furthermore, we used two strategies to evaluate the performance of DEGRN. Firstly, we used the above eight datasets as the inputs of DEGRN to train eight models, following the same pipeline with the above datasets. The AUROC was used as the metric for evaluating the performance differences between these eight models and DEGRN. Secondly, we used the DEGRN model constructed on E-GEOD-141730 and GSE80744 to predict the gene interactions when using the expression data of these eight datasets as the inputs. The AUROC value and F1 score were used as the metrics of comparison. The F1 scores were calculated as follows:

$$Precision=\frac{TP}{TP+FP}$$

$$Recall=\frac{TP}{TP+FN}$$

$$F1\;score=2\times \frac{Precision\times Recall}{Precision+Recall}$$

### 4.4. Prediction of Gene Function for TFs by DEGRN

The GO annotation of each gene was downloaded from The Arabidopsis Information Resource (TAIR) [80]. For each TF, the target gene obtained by DEGRN was subjected to GO enrichment analysis using the hypergeometric distribution in ClusterProfiler v4.0 [81]. The *p*-value was adjusted with the Benjamini–Hochberg (BH) method, and the *P*-adjust-value cutoff was set as 0.05 to give the final results. Only experimental and curated GO biological process annotations were considered (version August 2018). GOs corresponding to terms located at the root of the GO hierarchy were excluded (GO:0008150, GO:0009987, GO:0008152, GO:0044237, GO:0071704, GO:0050896, GO:0065007, GO:0032502, GO:0050789, GO:0032501, GO:0007275, GO:0050794, GO:0006355, GO:0045893, GO:0045892), as previously reported [27]. The recovered TF of GO was defined as the TF containing a certain GO term and that was successfully enriched for the same GO term by its predicted target genes. The recovery of the GO term was defined as the number of TFs successfully recovered divided by the number of TFs with the corresponding GO term. 

### 4.5. Comparison between Differentially Expressed Genes (DEGs) and DEGRN

Time-course expression data of a control and cold treatment of Arabidopsis were downloaded from the GEO database with IDs GSE5620 and GSE5621 [82]. The differentially expressed genes (DEGs) were identified with “limma” packages. We calculated the corresponding times between the control and cold treatment, including 0.5 h, 1.0 h, 3.0 h, 6.0 h, 12.0 h, and 24.0 h. 

### 4.6. Validation of Leaf Senescence Using Time-Course Data

The expression data for the lifespan of Arabidopsis leaves were obtained from GSE43616 in the GEO database. This time-course dataset contains the leaves of Arabidopsis at different developmental stages, set at 2-day intervals from 4 to 30 days. The heatmap of the genes predicted from DEGRN was constructed using the R package “pheatmap”. The SAGs were randomly selected from the LSD database [63]. 

### 4.7. Plant Materials and Growth Conditions

The transfer DNA (T-DNA) insertional mutant *maf5* was obtained from the Nottingham Arabidopsis Stock Center (NASC). Seeds were surface-sterilized in 10% (*v*/*v*) sodium hypochlorite for 10 min, washed 3 times with sterilized water, and then grown on Murashige and Skoog medium plus 3% sucrose and 0.6% agar (PH5.8) after 3 days of vernalization in darkness at 4 °C. The 7-day-old seedlings were transferred into soil and were grown at 22 °C in a 16 h light/8 h dark cycle. The selection of four stages of a leaf was described in a previous study [83]. The total RNA was isolated from the seedlings with TRIzol reagent. qRT-PCR analysis was performed with a Roche Light Cycler 480 real-time PCR system using SYBR Green Master Mix (Vazyme Biotech Co., Ltd., Nanjing, China). Actin2 was used as an internal control for data normalization.

### 4.8. Transcriptome Analysis Based on MAF5 Expression in 1001 Arabidopsis

To investigate the potential mechanism of *MAF5*, we sorted the samples with the expression of MAF5 in 1001 Arabidopsis (GEO ID: GSE43858). We selected the 4 accessions with no expression of MAF5 (6921, 763, 6931, and 6982) and 6 accessions with the highest expression of MAF5 (6961, 8264, 7342, 7068, 6989, and 6994), which had a TPM value of more than 20. The DEGs were filtered with the criteria of “|logFC| > 1 and *p* value < 0.05”. GO and KEGG enrichment analyses were conducted using ClusterProfiler in R [81]. 

### 4.9. Comparison with Previously Reported Methods 

To ensure a fair comparison was made, the GO enrichment of target genes per TF from the other three methods was obtained in the same way (i.e., using ClusterProfiler v4.0) [81]. The background GO file was acquired from the Arabidopsis Information Resource (TAIR) [80]. Only the top 50 TFs predicted for abiotic stress were selected for the comparison between DEGRN and the other three methods. The performance of each method was defined as the number of known regulators recovered. The information on the TF family in Arabidopsis was downloaded from PlantTFDB [84]. 

## 5. Conclusions

In this study, we developed a model named DEGRN, which can merge the expression data of bulk RNA-Seq and scRNA-Seq. We used DEGRN to investigate transcription factors and their interactions and, in this way, we predicted novel potential functions of transcription factors. Taking leaf senescence as an example, we obtained a set of novel transcription factors that may be involved in leaf senescence and validated the potential role of MAF5 through transcriptomics analysis and phenotype analysis. Overall, the comprehensive transcriptional regulation predicted by DEGRN can provide a valuable basis for further investigations into gene functions and may find breeding applications. 

## Figures and Tables

**Table 1:** Comparison between DEGRN and the experimental evidence.

Comparison	Overlap Numbers	Expected Overlap of Permutation Test with 10,000 Replicates	Range	*p*-Value	Enrichment Fold
DEGRN vs. ChIP genes	1245	687	456–878	*p* < 1 × 10^−4^	1.81
DEGRN vs. DE genes	2890	1393	1032–1703	*p* < 1 × 10^−4^	2.07
DEGRN vs. Y1H data	586	194	154–234	*p* < 1 × 10^−4^	3.02

## Data Availability

All data used in this study are available within the paper and within its Supplementary Materials published online.

## Supplementary Materials

The following supporting information can be downloaded at: https://www.mdpi.com/article/10.3390/plants13091276/s1, Figure S1: The overview of DEGRN scores; Figure S2: The results of DEGRN on different combinations of bulk transcriptome data and single-cell transcriptome data; Figure S3: Examples of novel networks related to development, including root, leaf, anther, and pollen; Figure S4: Examples of novel networks related to fatty acid metabolism and stress responses, including UV, salt, and metal ions; Figure S5: The enrichment analysis is based on the differentially expressed genes (DEGs) between MAF5^−^ and MAF5^+^; Table S1: Details of 20 repeats of 10-fold cross-validation (CV); Table S2: A comparison between the TF target genes derived from the predictor and those identified from DAP-seq analysis; Table S3: The details of a network of leaf senescence; Table S4: Overview of number of target genes predicted from DEGRN, EXPLICIT, iGRN, and AtRegNet; Table S5: Overview of a number of target genes for different TF families; Table S6: Comparison of known regulators associated with abiotic stresses of from DEGRN, EXPLICIT, iGRN, and AtRegNet; Table S7: The details of the literature for ChIP genes and Y1H genes; Table S8: The details of different dataset combinations.
