# Supplementary material for: Identification of Novel Regulators of Leaf Senescence Using a Deep Learning Model
Source: Plants (Basel). 2024 May 5;13(9):1276. doi: 10.3390/plants13091276 (PMC11085074; doi:10.3390/plants13091276)
Supplement: Supplementary file 1 [file plants-13-01276-s001.zip › plants-2960824-supplementary/Supplemental_Figures.pdf]

Supplemental Figures

Supplemental Figure S1.

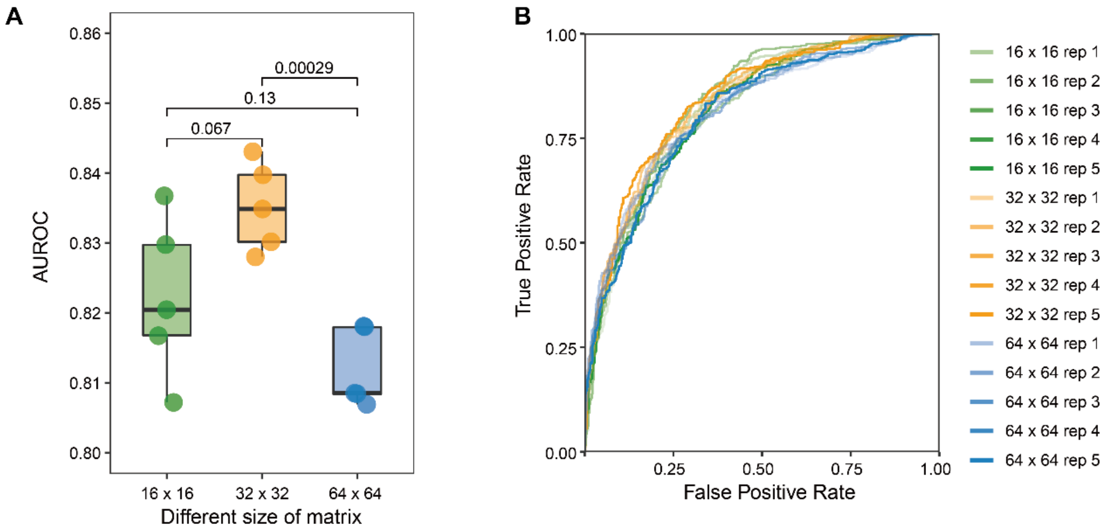

Supplemental Figure S1. The overview of DEGRN scores. (A) Boxplot of AUROC scores among different bin sizes. (B) The Receiver Operating Characteristic (ROC) plot among different bin sizes.

Supplemental Figure S2.

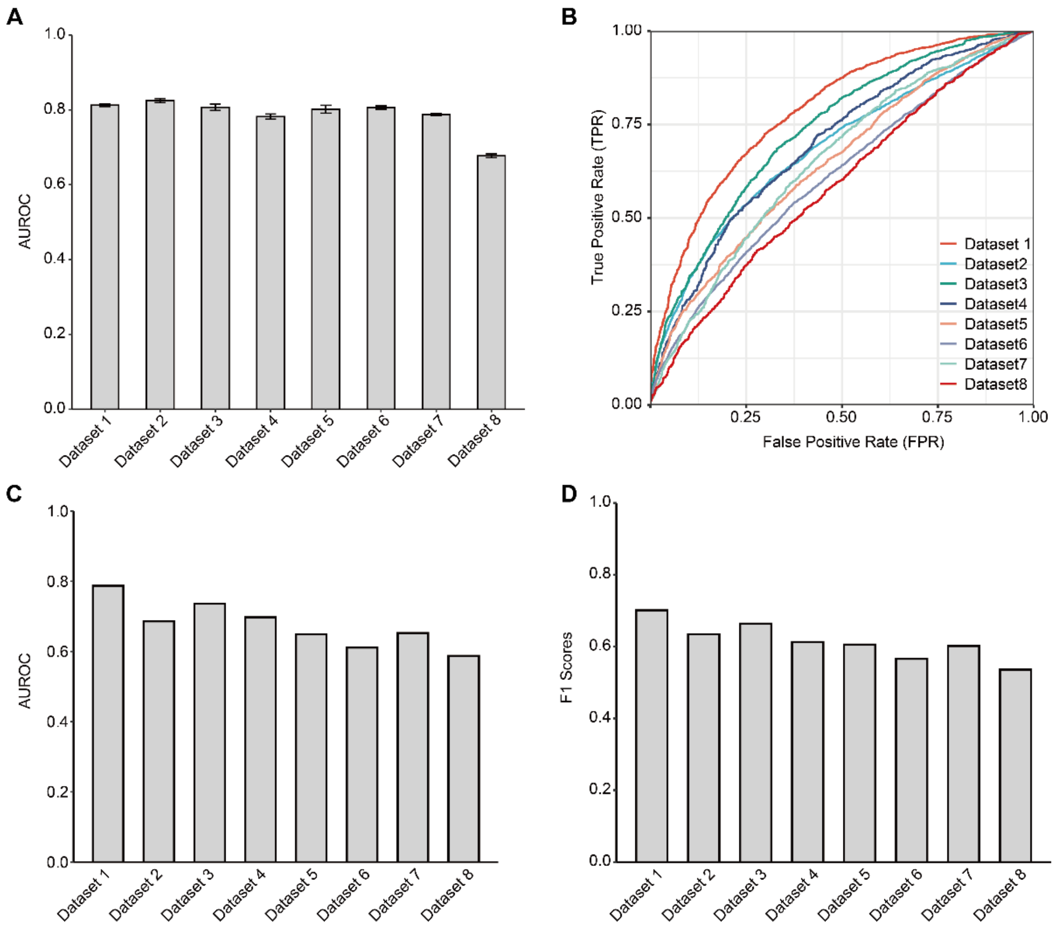

Supplemental Figure S2. The results of DEGRN on different combinations of bulk transcriptome data and single-cell transcriptome data.

(A) AUROC scores of the models built by the dataset in the first strategy. (B) Receiver Operating Characteristic (ROC) plot of eight models in (A).

(C) and (D) indicate the AUROC and F1 scores using the 8 datasets as the evaluation data.

Supplemental Figure S3.

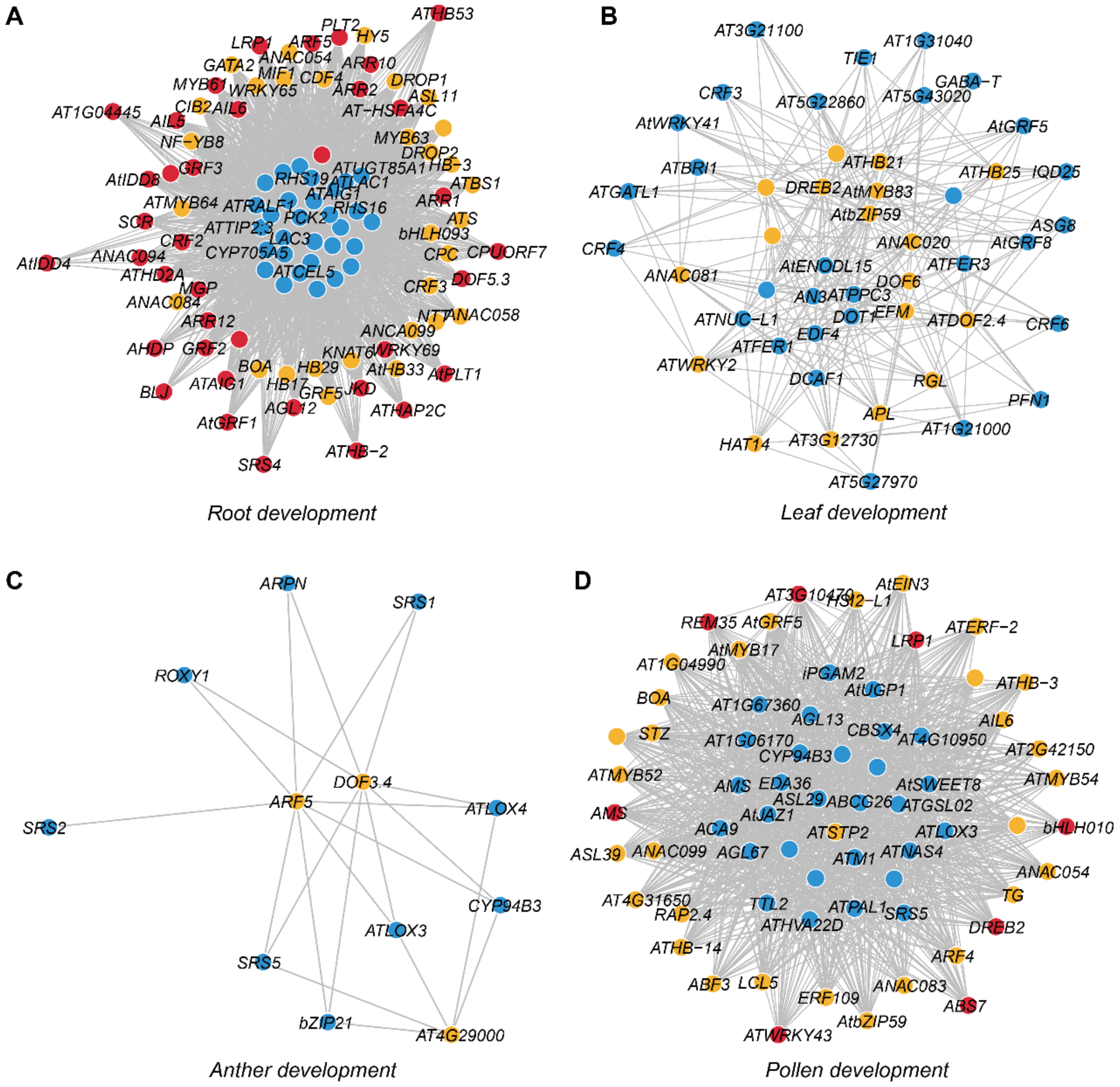

Supplemental Figure S3. Examples of novel networks related to development, including root (A), leaf (B), anther (C), and pollen (D). The red dots represent the known TFs that had been previously reported; the yellow dots represent the novel TFs predicted by DEGRN; and the blue dots represent the target genes obtained by DEGRN; the grey lines represent the correspondence of TF and its target genes which was enriched in the BP.

Supplemental Figure S4.

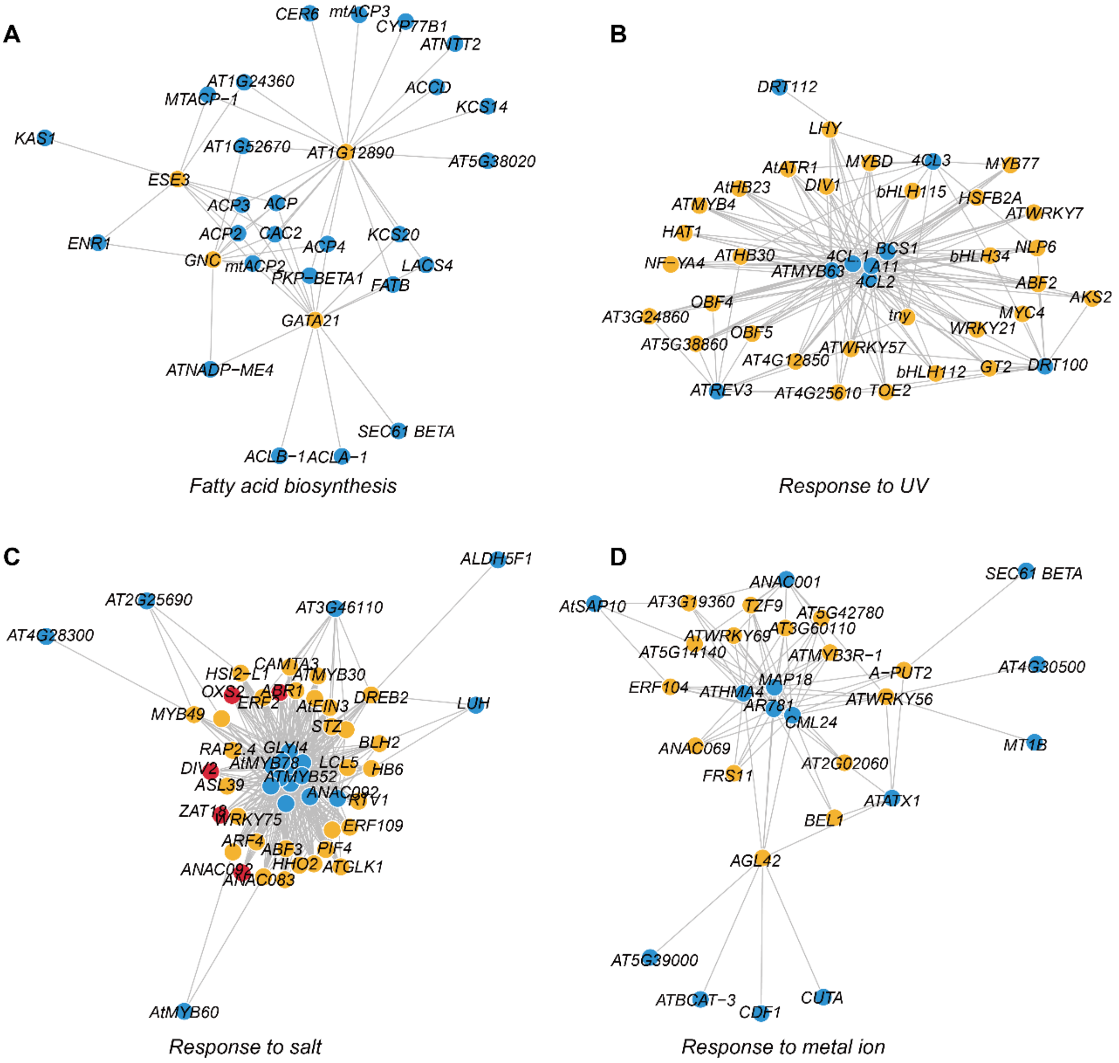

Supplemental Figure S4. Examples of novel networks related to fatty acid metabolism (A) and stress responses, including UV (B), salt (C), and metal ions (D). The red dots represent the known TFs that had been previously reported; the yellow dots represent the novel TFs predicted by DEGRN; and the blue dots represent the target genes obtained by DEGRN; the grey lines represent the correspondence of TF and its target genes which was enriched in the BP.

Supplemental Figure S5.

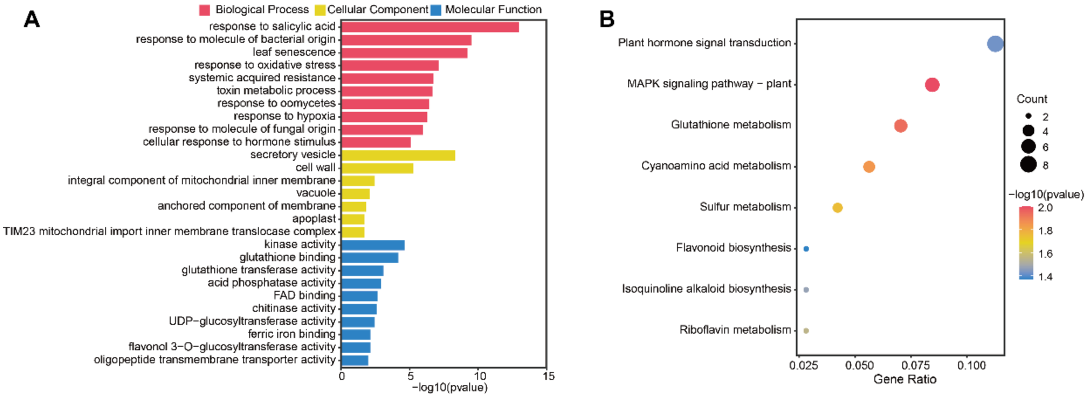

Supplemental Figure S5. The enrichment analysis is based on the differentially expressed genes (DEGs) between MAF5$^-$ and MAF5$^+$. (A) and (B) represent the results of Gene Ontology (GO) and KEGG.
